# Editorial: Advanced Neuroimaging of Brain Metastases

**DOI:** 10.3389/fneur.2021.668310

**Published:** 2021-03-17

**Authors:** Behroze A. Vachha, Susie Y. Huang, Tarik F. Massoud

**Affiliations:** ^1^Department of Radiology, Memorial Sloan Kettering Cancer Center, New York, NY, United States; ^2^Department of Radiology, Massachusetts General Hospital and Harvard Medical School, Boston, MA, United States; ^3^Department of Radiology, Stanford University, Stanford, CA, United States

**Keywords:** brain metastases, advanced neuroimaging, radiomics, imaging biomarkers, SWI

Metastasis is the most common cause of brain cancer. Advanced magnetic resonance imaging (MRI) and positron emission tomography (PET) techniques are playing an increasingly important role in the surveillance, diagnosis, and management of patients with brain metastases as treatments for systemic cancers continue to improve. These advanced neuroimaging methods enable better delineation of metastatic lesions, while also providing a rich array of information regarding the microstructural, vascular, metabolic, and functional properties of metastases and the affected brain parenchyma. However, barriers remain in the widespread translation and adoption of these techniques. The challenges of imaging patients with brain metastases include the heterogeneity of primary tumors and the tumor microenvironment. The varied appearances of metastases before, during and after treatment, especially in the face of a growing arsenal of new cancer therapies, are further difficulties to contend with.

Advanced neuroimaging biomarkers promise to improve the non-invasive characterization, prognostication, and evaluation of treatment response and post-treatment effects related to brain metastases. Such biomarkers may aid in the differentiation of metastases from other entities and serve as valuable adjuncts to conventional imaging in distinguishing disease progression from treatment-related effects. The standardization and validation of advanced imaging biomarkers will need to be addressed to facilitate the adoption of such techniques in clinical trials and clinical practice. A survey of the available techniques will help to define more precisely the role of state-of-the-art imaging approaches in targeted problem-solving, as well as their broader utility in elucidating the underlying cancer biology.

This Research Topic provides a glimpse of current and emerging advanced neuroimaging techniques used in the care of patients with brain metastases. Tong et al. provide a comprehensive review of advanced MRI techniques for brain metastases. These techniques are useful for both diagnosis, including differentiating between types of malignancy, and assessment of treatment response, including distinguishing radiation necrosis from disease progression. These methods include: black blood MRI; magnetic resonance spectroscopy; quantitative magnetization transfer imaging; dynamic contrast-enhanced MRI to measure the transmembrane water exchange rate; chemical exchange saturation transfer measurement; perfusion imaging; and radiomics and artificial intelligence techniques.

Picking up on the radiomics theme, Lohmann et al. review radiomic techniques and features when applied to PET/MRI of brain metastases. They discuss the underlying rationale of radiomic analysis, which is to identify appropriate characteristic image features and to use these to generate predictive or prognostic mathematical models. Hence, radiomics of brain metastases can be considered as a tool to complement established imaging analysis methods and other clinical measures that can be jointly used to make treatment decisions or final diagnoses with improved confidence.

Goncalves Filho et al. studied a highly accelerated Wave-controlled aliasing in parallel imaging (Wave-CAIPI) post-contrast 3D T1 SPACE MRI sequence for brain metastases, and found that it provides equivalent visualization of lesions and an overall diagnostic quality, with three times reduced scan time compared to standard 3D T1 SPACE MRI.

Two articles present the role of susceptibility weighted imaging (SWI) MRI for brain metastases. Schwarz et al. review its role in early diagnosis, determination of type of malignancy, and treatment monitoring, and they discuss therapy-associated changes that can affect SWI. They also review recent insights on the role of “isolated SWI signals” and the controversy on the specificity of SWI for early detection of brain metastases. Ceballos-Ceballos et al. compared unenhanced SWI and gadolinium-enhanced SWI (SWI-Gd) to assess if the latter improves brain metastasis detection in combination with other MRI sequences. They found that SWI-Gd may improve the diagnostic yield of brain metastases.

Ari Wijetunga and Jonathan Yang provide useful insights into various aspects of diagnostic neuroimaging that radiation oncologists rely on for clinical decision-making, radiation treatment planning, and assessment of treatment response or complications of brain metastases. Also of relevance to radiation treatment of brain metastases, Swinburne et al. conducted a study to determine if early intratumoral changes in interstitial fluid pressure (IFP) and velocity (IFV), estimated from computational fluid modeling using dynamic contrast-enhanced MRI, can predict long-term outcomes of lung cancer brain metastases treated using stereotactic radiosurgery. They found that early post-treatment assessment of IFP and IFV can be used to predict long-term response of lung cancer brain metastases to radiosurgery, allowing timely treatment modifications.

Finally, Kalakoti et al. present an interesting analysis of morphometric and volumetric differences across anatomical brain regions in patients with metastases presenting with seizures. That information could provide useful biomarkers in the identification of seizure expression and could serve as a neuronal target for mitigation. Using brain segmentation of T1-MPRAGE MRI images, they found that certain brain regions, such as the pars orbitalis, supramarginal and temporal gyrus (middle, transverse), and the pre-cuneus contribute a maximal potential for differentiation of seizure from non-seizure patients.

Overall, this collection of research articles and up-to-date reviews might act as a useful platform of knowledge for scientists and clinicians grappling with the difficult management of brain metastasis patients. We believe that the next big challenges in management of these patients will be to scale up various efforts and tools in the field of advanced neuroimaging of brain metastases. For instance, this will require new strategies and advances in techniques that include diffusion, perfusion, spectroscopy, functional MRI, positron emission tomography, radiomics and machine learning, to name a few. Moreover, in this era of precision medicine, these advanced neuroimaging techniques will need to be integrated with molecular information to allow personalized targeting of each patient's unique type of metastatic tumor.

## Author Contributions

All authors contributed to manuscript revision, read, and approved the submitted version.

## Conflict of Interest

The authors declare that the research was conducted in the absence of any commercial or financial relationships that could be construed as a potential conflict of interest.

